# Situation Analysis and Recommendations for the Biosecurity Status of Dairy Farms in Punjab, India: A Cross-Sectional Survey

**DOI:** 10.3390/ani13223458

**Published:** 2023-11-09

**Authors:** Pankaj Dhaka, Ilias Chantziaras, Deepthi Vijay, Manmeet Singh, Jasbir Singh Bedi, Nele Caekebeke, Jeroen Dewulf

**Affiliations:** 1 Veterinary Epidemiology Unit, Faculty of Veterinary Medicine, Ghent University, Salisburylaan 133, 9820 Merelbeke, Belgium; ilias.chantziaras@ugent.be (I.C.); nele.caekebeke@biocheckgent.com (N.C.); jeroen.dewulf@ugent.be (J.D.); 2 Centre for One Health, College of Veterinary Science, Guru Angad Dev Veterinary and Animal Sciences University, Ludhiana 141004, India; deepthivijay@kvasu.ac.in (D.V.); meet.mehn@gmail.com (M.S.); bedijasbir78@gmail.com (J.S.B.); 3 Department of Veterinary Public Health, College of Veterinary and Animal Sciences, Kerala Veterinary and Animal Sciences University, Mannuthy, Thrissur 680651, India

**Keywords:** biosecurity, buffaloes, cattle, dairy, herd management, India

## Abstract

**Simple Summary:**

Farm biosecurity is crucial for preventing infections and maintaining animal health. The present study assessed biosecurity practices on dairy farms in Punjab, India, using a standardized scoring system. The study found that the mean external and internal biosecurity scores for the selected dairy farms were 45.4% and 43.7%, respectively. Among the different aspects of biosecurity, farms performed best in vermin control and adult cattle management, while they scored lowest in purchase and reproduction, and health management. Interestingly, larger farms tended to have better biosecurity. This study highlights the need for improvement in biosecurity measures on dairy farms, particularly in the areas of purchasing animals and health management. This research underscores the importance of addressing these issues to enhance animal health and reduce disease risk in the region. Further investigations are recommended to better understand the factors influencing biosecurity practices on these farms.

**Abstract:**

Farm biosecurity is an important herd management strategy to assure infection prevention and animal health. The present study aimed to evaluate the implementation of biosecurity measures on dairy farms in Punjab, India, using the standardized Biocheck.UGent™ scoring system. Convenience sampling was used to select 94 dairy farms, comprising both cattle and buffaloes, with a mean herd size of 74.8 animals. The study found that the mean external and internal biosecurity scores for the selected dairy farms were 45.4% and 43.7%, respectively. Among the subcategories, the highest external biosecurity score was observed for ‘vermin control and other animals’ (63%), and the highest internal biosecurity score was observed for ‘adult cattle management’ (76.6%). Whereas the lowest score for external biosecurity was observed for ‘purchase and reproduction’ (30.6%), and the lowest score for internal biosecurity was observed for ‘health management’ (33.6%). The overall mean biosecurity score of the present study was 44.8%, which was lower than the overall mean global score of 52%. The correlation analysis indicated a positive correlation between herd size and overall biosecurity scores, indicating that larger farms had, on average, higher biosecurity scores. Based on these results, it can be concluded that the implementation of biosecurity measures on dairy farms in Punjab needs improvement, especially in the subcategories of ‘purchase and reproduction’ and ‘health management’. Further research to identify the factors influencing the implementation of biosecurity measures on dairy farms in the region is recommended.

## 1. Introduction

Dairy farming is an important animal husbandry sector that contributes significantly to sustainable food production, livelihood, and economies worldwide [1]. Like other livestock sectors, dairy farms face significant risks from the introduction and spillover of infectious diseases, animal welfare concerns, and environmental contamination. These issues pose a threat to food security and safety, as well as cause socioeconomic losses for dairy farmers [2]. A number of infectious agents that affect dairy animals can also be transmitted to humans through direct contact or by consuming contaminated animal food products. This can result in the transmission of various zoonotic diseases [3]. Effective implementation of biosecurity measures is one of the important and widely used strategies to prevent and control the spread of infectious diseases on dairy farms [4].

Farm biosecurity is considered a critical component of One Health, and it involves implementing segregation, hygiene, or management procedures (excluding medically effective feed additives and preventive/curative treatment of animals) to reduce the likelihood of introducing, establishing, surviving, or spreading potential pathogenic agents to, within, or from a farm operation or geographical area [5]. Effective implementation of both external and internal biosecurity measures in dairy farms can significantly reduce the risk of disease transmission from infected animals, contaminated equipment, and people such as farm workers, visitors, and animal health professionals. This, in turn, can improve the health, productivity, reduction in antimicrobial usage, and welfare of the animals [6,7]. Studies have shown that, despite the significant role of biosecurity measures in preventing the spread of infectious diseases and the associated benefits, many dairy farmers lack awareness and knowledge with regard to the benefits and application of biosecurity measures [8]. 

Dairying is a crucial component of rural livelihood in India, with the Indian dairy sector being arguably the world’s largest agri-food sector, providing employment to over 70 million households [9,10]. In India, as per the 20th livestock census, the cattle population is recorded at 193.46 million, and the buffalo population is 109.85 million. These animals, respectively, contribute to 51% and 45% of the total milk production for the year 2021–22 [11]. In the state of Punjab, around 4 million buffaloes and 2.5 million cattle equally share in contributing 50% of the milk production. The region primarily features mixed-type dairy farms (having both buffalo and cattle), with a significant presence in small and medium-sized operations located in rural and peri-urban areas [12]. However, more than half of India’s dairy sector remains informal, highlighting the need for strengthening herd health management and techno-managerial approaches to move the sector toward sustainability [13]. 

India has been identified as a hotspot for emerging infectious diseases [14], and past epidemiological studies have reported the prevalence of various zoonoses associated with farm animals. Some of the important zoonoses include brucellosis [15], anthrax [16], toxoplasmosis [17], Q fever [18], and Crimean–Congo hemorrhagic fever [19]. The concerns have been raised by previous studies conducted in Punjab regarding the potential transmission of zoonotic diseases due to several risk factors and the limited awareness among livestock farmers in the region. Researchers observed that livestock farmers possess a low to medium level of knowledge about prevalent zoonotic diseases in the region [20]. The low level of education and being a cattle farmer were negatively associated with the farmer’s knowledge of zoonotic diseases [21]. Furthermore, it was noted that farmers’ adoption of biosecurity measures and the utilization of personal protective equipment by veterinary personnel were significantly lacking in the region [22]. This considerably increases the risk of zoonotic disease transmission and underscores the need for comprehensive assessments and awareness initiatives focused on farm biosecurity within the region.

Many times, the animal health sector in India mainly focuses on therapeutic measures and reactive responses toward infectious disease outbreaks [23]. The recent outbreak of lumpy skin disease (LSD) in 15 Indian states in 2022 resulted in significant socioeconomic losses due to bovine morbidity, mortality, and productivity losses [24]. Such outbreaks underscore the need to promote biosecurity management in the dairy sector to control and prevent the spread of emerging animal health threats. Therefore, it is essential to assess and quantify farm biosecurity practices to understand existing management practices and identify areas for improvement in the herd. In this context, the present study aims to assess the level of biosecurity measures in dairy farms in the Punjab state of India and formulate recommendations for adopting biosecurity measures in the study region.

## 2. Materials and Methods

### 2.1. Sampling Strategy

A cross-sectional survey was conducted using convenience sampling to recruit volunteer dairy farmers from Punjab. The outreach was facilitated through local contacts and an open call inviting participation in the study. Farm visits were scheduled between July 2021 and November 2022 based on the availability of farmers. A standardized procedure was followed during the visits to ensure uniformity in data collection, and participating farmers completed a farm biosecurity questionnaire. The surveys were carried out on-site, with interviewers simultaneously observing the biosecurity measures in place during the farm visit. The interviewer also observed the ongoing biosecurity measures during the herd visit. Verbal consent was obtained from the farm owners to use the farm data anonymously for academic and research purposes.

### 2.2. Ethical Statement

The research was conducted in accordance with the Declaration of Helsinki and national standards. All the required ethical considerations were taken into account. The nature of the study was completely voluntary, and informed consent was obtained from study participants. The details of the participants were anonymous, and data confidentiality was properly maintained.

### 2.3. Data Collection and Biosecurity Quantification

The farm visits were conducted to assess the implementation of biosecurity measures using the Biocheck.UGent™ (Ghent, Belgium) questionnaire. This is a risk-based assessment tool that allows for the quantification and comparison of the biosecurity status of herds within and between countries [25]. The dairy cattle survey includes a maximum of 124 questions, and the survey results in a score from 0 to 100 for both external and internal biosecurity subcategories. In the evaluation process of the survey, responses that described the ideal biosecurity scenario consistently received full points for the question, whereas less favorable answers were assigned partial or no points. In most instances, only two scoring options were applicable, indicating the presence or absence of a specific measure. However, in a few cases, an intermediate score was used. Notably, for certain questions that originally offered three or more response options (e.g., always, sometimes, and never), a simplified scoring approach was adopted, with the intermediate answer (e.g., sometimes) being treated on par with the lowest-scoring response [26]. The final score for internal and external biosecurity is the weighted average of the subcategory scores. A score of 0 represents an ‘absolute lack of any biosecurity measures’, while a score of 100 indicates ‘full application of all assessed biosecurity measures’ (Source weblink: https://biocheckgent.com/en/questionnaires/dairy-cattle, accessed on 14 March 2021). Damiaans et al. (2020) provide a more detailed description of the Biocheck.UGent™ system for the dairy cattle [26].

The dairy cattle section of Biocheck.UGent™ consists of five subcategories for external biosecurity ((a) purchase and reproduction, (b) transport and carcass removal, (c) feed and water, (d) visitors and farm workers, and (e) vermin control and other animals) and six subcategories for internal biosecurity ((a) health management, (b) calving management, (c) calf management, (d) dairy management, (e) adult cattle management, and (f) working organization and equipment). 

To ensure that the survey questions were easily understood by the dairy farmers, they were translated into the local language (Punjabi or Hindi) as per the requirement. The data collected through the survey were then transferred to the online system of Biocheck.UGent™ (www.BiocheckGent.com) for calculation of the biosecurity scores and further analysis.

### 2.4. Statistical Analysis

The descriptive statistical analysis for the distribution of responses was carried out using Microsoft^®^ Excel 2019 (Microsoft Corp., Santa Rosa, CA, USA). The mean, median, standard deviation, and minimum and maximum scores were obtained for each subcategory. The normal distribution of the data was assessed by using a Kolmogorov–Smirnov test. The possible associations between herd size and overall biosecurity scores and external and internal biosecurity scores were evaluated by means of correlation analysis. The correlation coefficient was categorized into weak (0–0.25), fair (0.25–0.5), good (0.5–0.75), and excellent (0.75–1), based on the R-value as described by [27]. The statistical significance level was set to *p* < 0.05. Further, to compare biosecurity scores among distinct farm types, namely buffalo farms, cattle farms, and mixed cattle and buffalo farms, Tukey’s HSD (honestly significant difference) method was employed for pairwise comparisons. The F statistic initially determined overall mean disparities, and the subsequent application of Tukey’s HSD test identified statistically significant differences between various pairs of means, if present.

## 3. Results

### 3.1. Herd Characteristics

**Figure 1:**
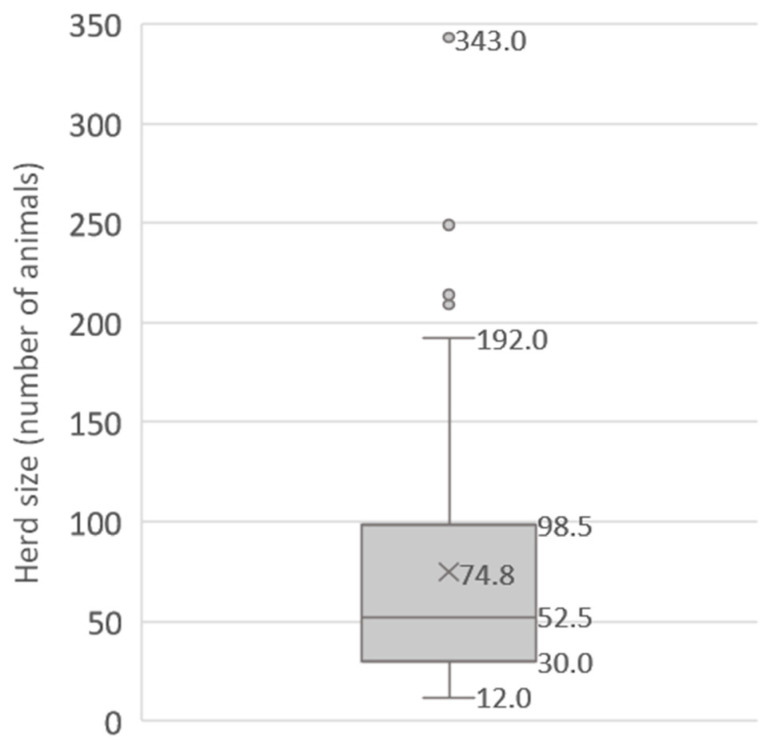
Distribution of the studied farms by number of animals.

A total of 94 commercial dairy farms, comprising both cattle and buffaloes, were enrolled in the study. Out of the 94 farms, 44 farms exclusively had cattle, 39 farms had a mix of buffaloes and cattle, and 11 farms solely had buffaloes. The mean herd size of the participating farms was 74.8 bovines (SD = 65.5; range = 12–343). Figure 1 shows the distribution of the selected dairy farms according to their herd size. The median herd size was 52.5 animals, and 75% of the farms (below the upper quartile) had less than 100 animals.

### 3.2. Biosecurity Scoring of Dairy Farms and Comparison with Global Mean Scores

**Figure 2:**
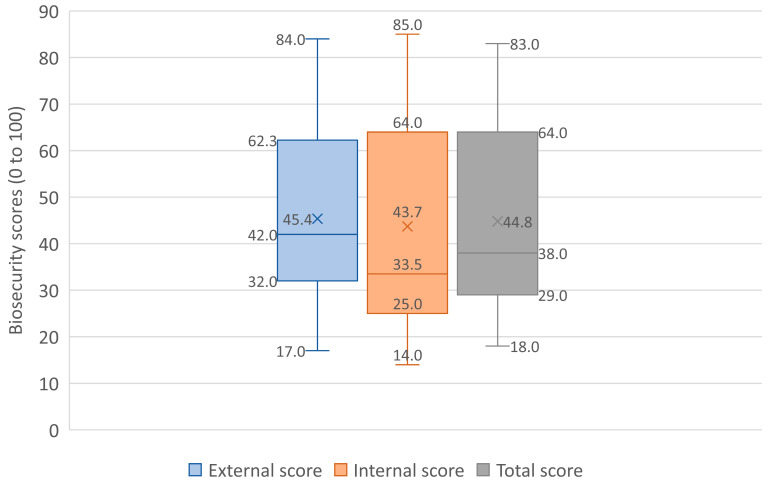
Comparison of external, internal, and total biosecurity scores among the study farms.

The mean external biosecurity score for the selected dairy farms was observed to be 45.4% (SD = 18), with a median of 42% and a range of 17–84%. Among the different subcategories, the highest mean score was observed for ‘vermin control and other animals’ (63%), followed by ‘feed and water’ (61%), ‘transport and carcass removal’ (52.9%), and ‘visitors and farm workers’ (47.6%), and the lowest score was observed for ‘purchase and reproduction’ (30.6%). Comparing the mean scores with the global average (as provided by Biocheck.UGent™), the subcategories ‘transport and carcass removal’, ‘feed and water’, and ‘vermin control and other animals’ had higher mean scores in the present study farms. However, the overall global external biosecurity mean score was higher than the mean score of the present study (67 versus 45.4) (Table 1; Figure 2).

The mean internal biosecurity was 43.7% (SD = 22.4), with a median of 33.5% and a range of 14–85%. Among the subcategories, the highest score was observed for ‘adult cattle management’ (76.6%), followed by ‘dairy management’ (55.1%), ‘calving management’ (42.4%), ‘working organization and equipment’ (41.9%), ‘calf management’ (41.6%), and the lowest score was observed for ‘health management’ (33.6%). Comparing the mean scores with the global average, except for ‘calf management’, the mean scores of all other subcategories were higher in the present study. Additionally, the overall internal biosecurity mean score was higher than the global mean score (43.7 versus 37) (Table 1, Figure 2). However, the overall mean biosecurity score of the present study was 44.8%, which was lower than the overall mean global score of 52%.

### 3.3. External Biosecurity

The results with regard to purchase and reproduction suggest a lack of focus on animal health assessment during the procurement of new animals. Although farmers usually acquire animals from familiar sources, diagnostic testing for endemic diseases is not commonly conducted. Only a small proportion (2.1%) of farmers reported testing the milk of newly introduced animals, primarily for suspected mastitis cases. Also, only 24.5% (*n* = 23) of the farmers adhered to the quarantine protocol for newly introduced animals, and there is no uniformity in terms of the duration of quarantine (the average duration observed was 9.7 days). The quarantine protocols are also not being adequately followed during the reintroduction of animals into herds. A total of 27.6% (*n* = 26) of farmers indicated that their animals leave their farms for animal shows and markets, but over half (53.8%, *n* = 14) of these farmers neglect to keep their animals in quarantine upon returning to the herd. Many farms (61.7%, *n* = 58) use artificial insemination as a reproductive tool, whereas 36 farms (38.3%) were still found to rely on a bull service for breeding, but only 22.2% (*n* = 8) of these farms conduct regular screening of the bulls, mainly for endemic diseases such as brucellosis.

Among the surveyed farms, 31.9% (*n* = 30) reported performing transport baths, mainly involving the washing of tires for vehicles entering their premises. Only 19.2% (*n* = 18) of the farms indicated that the transport vehicles are consistently emptied upon arrival. On the other hand, a significant majority of farmers (80.8%, *n* = 76) acknowledged that animal transportation to their farms often occurs in vehicles not specific to their own farm and that other farm animals are frequently transported together. Moreover, only 16% (*n* = 15) of the farms indicated they regularly cleaned and disinfected the transport vehicle prior to entry onto the farm.

More than half (55.3%, *n* = 52) of the farms lacked separate carcass storage space with a hard surface floor. A substantial proportion (30.9%, *n* = 29) of farmers frequently spread manure from other farms on their farmlands and pastures. While about 60% (*n* = 56) of farmers protected their feed facilities from pets, over half (56.4%, *n* = 53) admitted that pet dogs and/or cats could access their stables. 

A total of 30.8% (*n* = 29) of farms required visitors to notify them before visiting. Regular compliance with protocols for farm-specific clothing and boots for workers and visitors was observed in 26.6% of the farms. Furthermore, over half of the farms (53.2%, *n* = 50) did not provide separate areas for changing clothes, washing hands, and boots. 

Only 22.3% (*n* = 21) of farmers focused on insect control programs (mainly flies and mosquitoes), and only one farm was implementing bird control programs with nets. A total of 24.5% (*n* = 23) of farms indicated that their animal has access to outside natural water bodies, whereas 30.8% (*n* = 29) expressed the possibility of contact of their farm animals with animals from other farms.

### 3.4. Internal Biosecurity

With regard to animal health management, 31.9% (*n* = 30) of farms routinely isolated sick animals from healthy groups, with the majority (63.8% out of 31.9%) of these farms separating animals only when clinical signs were evident. Individual maternity pens or C-section boxes were provided in 10.6% (*n* = 10) of farms, while many farms (51.1%, *n* = 48) used shared maternity pens. Most farms (87.2%, *n* = 82) implemented proper cleaning and disinfection procedures for used obstetric materials before and after each calving or abortion; however, the remaining farms followed a cleaning protocol and only applied disinfection when handling cases of abortions. The practice of testing animals after abortion was observed to be low, with only 3.2% (*n* = 3) of farmers conducting the test routinely. However, 93.6% of farmers acknowledged the importance of testing if an animal has repeated abortions or if their veterinarian recommends it. Most of the farms (97.9%, *n* = 92) disposed of fetal membranes and tissues away from the farm premises (on the manure pile/slurry pit) after a calving or abortion.

A total of 62.8% (*n* = 59) of farms were found to follow the work line of taking care of sick animals after attending to the healthy animals, whereas 30.8% (*n* = 29) of the farms were not following any specific order in this context. Only 35.1% (*n* = 33) of farms maintained animal health records, and 41.5% (*n* = 39) had written protocols for vaccination, disease treatment, and hygiene procedures. A total of 33% (*n* = 31) of farmers performed bacteriological testing of farm water. However, they preferred to conduct such testing only when the farm faced adverse health and production incidences, such as calf mortalities, diarrhea, and abortion outbreaks. 

Except for nine farms, the others were following the administration of colostrum during the first six hours of birth. However, the assessment of colostrum quality is not commonly practiced. A total of 35.1% (*n* = 33) of the farms reported housing the calves in individual calf boxes/hutches or separate areas. The majority of the farms (77.7%, *n* = 73) regularly cleaned the feeding buckets after each feeding session. The concept of frozen colostrum or an artificial reserve of colostrum was not applied by any farm in the study.

The manual milking method was the primary approach utilized for milking, accounting for 85.1% (*n* = 80) of the farms employing this technique. Among these farms, more than half (51.1%; *n* = 48) adopted a wet cleaning technique for the teats, followed by drying them with separate towels. A total of 61.7% (*n* = 58) of farms were examining the milk during fore-stripping. Except for seven farms, the others were keeping the animals upright for 30 min to one hour after milking. Furthermore, the majority of farms (83%; *n* = 78) followed the practice of milking cows with mastitis and/or a high somatic cell count (SCC) last in the milking order. Half of the farms (50%; *n* = 47) were clipping udders twice or more per year, whereas 30% (*n* = 28) were clipping at least once a year. Only 39.4% (*n* = 37) of the farms were carrying out regular (i.e., minimum once per year) bacterial examination of the udders of all animals. The average frequency for cleaning and disinfection of an adult animal stable was listed to be 3.8 and 3.4 times per year, respectively. 

A total of 45.7% (*n* = 43) of the farms were grouping the animals as per ‘age category’ in the stable. Only 35.1% (*n* = 33) of the farms were regularly following hoof disinfection footbath for adult animals. The farm work line in the order of handling youngest to oldest animals was followed by 55.3% (*n* = 52) of the farms. Most of the farms (96.8%; *n* = 91) were using their farm-specific equipment and not sharing it with other farms.

### 3.5. Correlation Analysis and Farm Type Comparisons 

**Figure 3:**
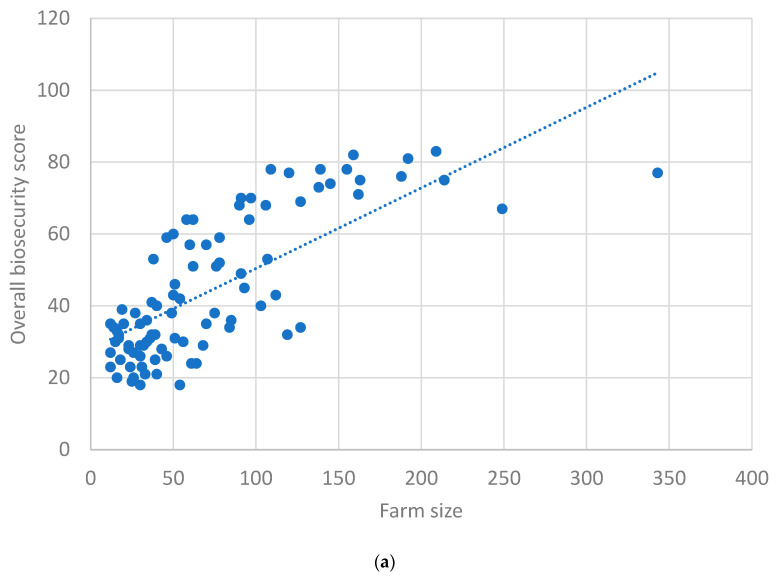

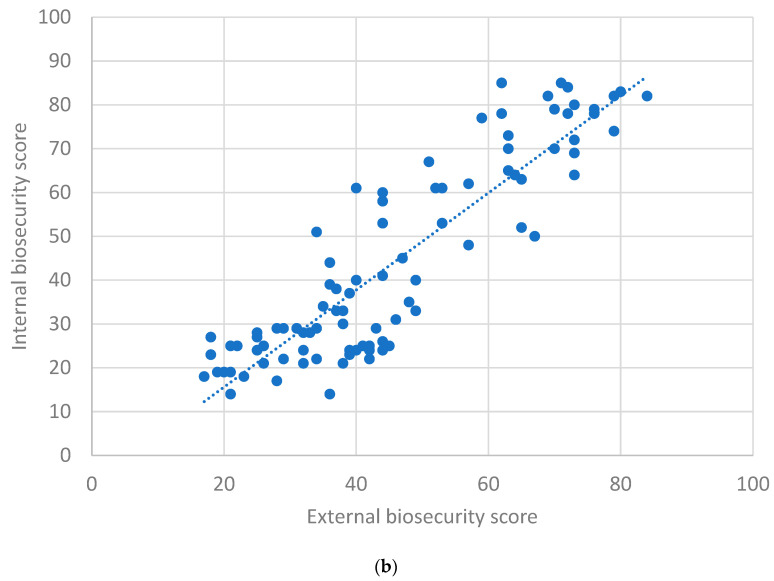
(**a**) Scatter plot between farm size and overall biosecurity scores. (**b**) Scatter plot between external and internal biosecurity scores.

Figure 3a depicts the scatter plot between herd size and overall farm biosecurity, for which Spearman’s correlation coefficient (r_s_) was found to be 0.75 (*p* < 0.01), indicating an ‘excellent’ positive correlation. Figure 3b depicts the scatter plot between internal and external biosecurity scores, for which r_s_ was observed as 0.84 (*p* < 0.01), indicating an ‘excellent’ positive correlation.

The analysis of biosecurity scores among farm types resulted in an F-ratio of 1.90, with a corresponding *p*-value of 0.15, indicating no statistical significance at *p* < 0.05. Similarly, pairwise comparisons of biosecurity scores among different farm types also revealed non-significant differences: buffalo farms versus cattle farms (*p*-value: 0.09), buffalo farms versus mixed farms (*p*-value: 0.14), and cattle farms versus mixed farms (*p*-value: 0.97).

## 4. Discussion

Farm biosecurity plays a crucial role in ensuring sustainable solutions to animal health, production, and welfare challenges [6,7]. However, limited data on dairy farm biosecurity parameters in India exist, and this is the first study in which the Biocheck.UGent™ scoring system is used to quantify these parameters. The Biocheck.UGent™ scoring system is a well-validated system that provides a comprehensive scoring of various farm biosecurity parameters, allowing for comparative analysis both within and between countries [28]. The Biocheck.UGent™ holds great potential for analysis of Indian farm biosecurity scoring and facilitating comparisons with other regions within the country and across the globe. Many of its parameters are well suited for Indian farm biosecurity conditions, enabling a comprehensive evaluation of biosecurity practices. The system can provide valuable insights into the strengths and weaknesses of biosecurity measures implemented in Indian farms, allowing for targeted improvements.

Punjab is the foremost commercial dairy farming region in India. Due to rapid urbanization and rising consumer income levels, there has been a growing demand for high-quality milk and milk products in the country. As a result, there is a noticeable transition occurring from traditional small-scale production to large-scale commercial production to meet these increasing demands [9]. Previous research has indicated that medium and large farmers are more aware and interested in adopting biosecurity interventions [29,30]. This explains the voluntary participation of the majority of the medium and large-scale dairy farmers in the study region.

The study observed that the overall external biosecurity mean score (45.4%) of the farms was lower than the global average (67%). This difference is mainly attributed to the low scores for the subcategories of ‘purchase and reproduction’ (30.6%) and ‘visitors and farmworkers’ (47.6%). The reason behind this could be attributed to the prevalent open herd system in most farms, where animals are frequently acquired from known sources without requiring any clinical proof of their health status. As a result, there are limited practices for testing newly purchased animals unless they show clinical signs. In line with this, a study conducted by Denis-Robichaud et al. (2019) revealed a lack of widespread adoption of biosecurity measures among Canadian dairy farms, with 48% of open herds lacking a strategy for introducing new and returning animals [31].

Further, the present study revealed that awareness about quarantine protocols and their implementation was low in the region. Recent studies have emphasized the importance of advocating the implementation of quarantine protocols as part of dairy farm biosecurity measures in order to prevent the introduction and spread of infectious diseases [32,33,34]. There has been low compliance with testing the maternal immunity of purchased calves, as it is commonly believed that these calves already possess sufficient maternal antibodies against endemic infections. Furthermore, the limited diagnostic facilities for endemic diseases were linked to lower compliance with animal and bull testing. A study by Damiaans et al. (2020) among dairy herds in Belgium found a higher score under the ‘purchase and reproduction’ category (39%) due to farms not purchasing cattle from external sources [26]. Farms that did carry out external purchases generally took precautions to limit possible disease transmission risks by following testing and quarantine procedures. However, in the present study, there was less emphasis on maintaining closed herds as animal purchase and sale among farmers was common. The low compliance for the use of laboratory diagnosis could be attributed to several factors, such as the unavailability of diagnostic facilities in the region, inadequate emphasis on adopting preventive procedures by veterinarians and/or farmers, high costs associated with diagnostic testing, and insufficient awareness of the benefits of early diagnosis of infectious diseases. Recent studies in the region have underscored the limited availability and adoption of diagnostic tools and preventive measures by animal health service providers. This, in turn, has contributed to the imprudent use of antimicrobial agents in the study region [35,36].

The low score for ‘visitors and farmworkers’ in terms of compliance with farm biosecurity measures can be attributed to several factors. Firstly, it is important to note that there is a prevailing socio-cultural norm where formal permission to visit farms is not considered important, so there is a general hesitancy toward seeking permission before visiting farms. Secondly, many farm workers are also engaged in work outside of the farm, which increases the risk of introducing diseases to the farm. Furthermore, there is a notable absence of adherence to farm-specific clothing and boots among farm workers, visitors, and animal health professionals. This can be attributed to a lack of awareness among farmers regarding the potential transmission of pathogens from contaminated clothing and boots worn by visitors. A previous study in the Punjab region found low compliance with personal biosecurity practices among the participant farmers, including limited use of personal protective equipment (PPE). Notably, handling the calving and dystocia cases often occurred without sleeves or gloves, and just 4% of surveyed farmers offered PPE to visitors. This highlights a concerning pattern of insufficient personal biosecurity measures within the agricultural community [22]. Studies have observed similar socio-cultural effects on biosecurity compliance in livestock systems across different regions of the world, where cultural beliefs hinder the effective implementation of biosecurity measures [37,38]. 

The mean internal biosecurity score (43.7%) was found to be higher than the global mean (37%), with scores above 50% observed for ‘adult cattle management’ and ‘dairy management’. This can be attributed to the large experience of the participating farmers in managing dairy farms and their emphasis on the fulfillment of biosecurity parameters associated with their farm boundaries (i.e., internal biosecurity), with less concern for external sources of disease. Moreover, farmers prioritize the health of their milking animals as they hold a high economic value, and the cost of treating diseases such as mastitis and abortions is substantial, in addition to the potential loss of production associated with these diseases. Therefore, they invest their best possible efforts in animal care and hygienic milk production. In a study by Denis-Robichaud et al. (2019) on Canadian dairy farmers’ perceptions and adoption of biosecurity practices, limited implementation was observed in both intra- and inter-herd measures aimed at minimizing infection spread. For example, 27% of respondents did not house sick or lame animals in their calving pens. Only 29% consistently ensured cow cleanliness before calving, and 27% regularly sanitized calving pens after each calving event. Additionally, fewer than 15% implemented measures to restrict or manage farm visitors [31].

The overall mean biosecurity score of the present study (44.8%) was lower than the overall mean global score (52%). This can be discussed in light of the fact that the majority of the farms that carried out the survey using Biocheck.UGent™ were from European regions where compliance with farm biosecurity is strongly advocated [26,39].

The positive correlation observed between herd size and overall farm biosecurity (rs: 0.75), as well as between internal and external biosecurity scores (rs: 0.84), reveals valuable insights. This suggests that, in our study context, as the herd size increases, the overall farm biosecurity tends to improve significantly. Past studies observed that herd size could be positively correlated with the economic condition and educational standard of farmers, which might lead to large farms adopting biosecurity measures more efficiently due to greater access to resources and awareness [29,40]. Similarly, the positive correlation observed between internal and external biosecurity scores highlights a strong association between the two aspects of biosecurity. This implies that farms excelling in one dimension of biosecurity are likely to perform well in the other as well, indicating a comprehensive approach to biosecurity implementation [41]. 

The analysis revealed that farm types (buffalo farms, cattle farms, and mixed cattle and buffalo farms) do not exhibit any significant difference in biosecurity practices in the study region. This uniformity may be attributed to the prevalence of mixed farming practices in the study region, where dairy cattle and buffaloes share similar management and biosecurity requirements. Additionally, regional factors and environmental conditions may contribute to the observed consistency in biosecurity scores across different farm types. Further research can explore the effectiveness of specific biosecurity measures within mixed farming regions to gain deeper insights into these practices. 

Although our study did not extensively assess the role of socio-cultural factors and field veterinarians in implementing farm biosecurity, previous research has highlighted their importance. For instance, a study on bovine tuberculosis in New Zealand demonstrated how farmers’ livestock purchasing behavior is influenced by the culture of their farm environment. While voluntary disease control schemes have been proposed to drive behavioral change, their success depends on understanding how different triggers work in different situations [42]. Additionally, many veterinarians in certain regions may be more focused on therapeutic approaches rather than preventive approaches. As noted by Preite et al. (2023), there is a need for training and improving biosecurity communication among farm veterinarians, as well as implementing formal regional risk mitigation programs through public–private collaboration to enhance biosecurity measures’ acceptance and maintenance on farms [43].

**Study recommendations:** To optimize biosecurity and mitigate disease risks on dairy farms, it is crucial for animal health professionals to educate farmers about the significance of implementing these biosecurity measures. By emphasizing the long-term benefits, such as improved farm productivity and enhanced animal health, professionals can encourage farmers to prioritize biosecurity practices. Policymakers and institutions should promote awareness and compliance through education and incentives while developing and enforcing regulations. The socio-cultural values of the local community should be considered when designing policies and interventions. The present study reflects the scope of improvement for implementation of quarantine protocols, awareness of carcass and placenta disposal, use of compartment-specific equipment, clothing, and boots, implementation of insect, bird, and rodent control measures, proper emphasis on calf management, and complying with biosecurity protocols for workers and visitors.

**Study limitations:** The study utilized a convenient sampling strategy by targeting volunteer farmers. However, it is crucial to note that this approach may introduce a potential bias known as ‘participant bias’. This is due to the possibility of including interested farmers with a higher level of knowledge about the targeted topics, leading to a skewed representation of the broader population. Despite this limitation, the study’s findings hold significance as there is limited knowledge or quantified data regarding dairy farm biosecurity in India. The results can help generate interest and awareness among the dairy chain stakeholders about the concept and implementation of farm biosecurity, laying a valuable foundation for future research in this field. 

## 5. Conclusions

This study highlights that the mean biosecurity scores for both external and internal factors on the selected dairy farms were below the global average. Key areas requiring improvement include ‘purchase and reproduction’ and ‘health management’. A positive correlation between herd size and overall biosecurity scores suggests that larger farms tend to have better biosecurity practices. The study clearly shows the need for carrying out a more quantitative assessment of the biosecurity practices in the region so that an evidence-based package of practices can be formulated. 

## Figures and Tables

**Table 1:** Biosecurity scores of the study farms and comparison with the global mean scores.

Biosecurity Parameter	Mean (%)	SD (%)	Median (%)	Min (%)	Max (%)	Global Mean (%) (*n* = 1848) *
**Total external biosecurity score**	**45.4**	**18**	**42**	**17**	**84**	**67**
(a) Purchase and reproduction	30.6	17.1	25	4	66	78
(b) Transport and carcass removal	52.9	28.4	52	0	100	47
(c) Feed and water	61	22.4	64	15	100	59
(d) Visitors and farmworkers	47.6	21.1	49	9	92	70
(e) Vermin control and other animals	63	25.2	65.5	0	96	62
**Total internal biosecurity score**	**43.7**	**22.4**	**33.5**	**14**	**85**	**37**
(a) Health management	33.6	32	18.5	0	93	31
(b) Calving management	42.4	22.1	27	11	91	31
(c) Calf management	41.6	20.2	34.5	10	82	43
(d) Dairy management	55.1	18.6	53	12	85	47
(e) Adult cattle management	76.6	17.2	75	28	100	40
(f) Working organization and equipment	41.9	31.1	17	3	100	38
**Overall biosecurity score**	**44.8**	**19.6**	**38**	**18**	**83**	**52**

* as of 21 February 2023.

## Data Availability

The data presented in this study are available on request from the corresponding author. The data are not publicly available due to privacy and confidentiality agreements with the participants.
